# Identification of VGLUT3-expressing LTMRs-recruited spinal circuits for itch inhibition

**DOI:** 10.1186/s13041-025-01245-3

**Published:** 2025-09-30

**Authors:** Xiaojing Su, Liangbiao Wang, Xiaoqing Liu, Yan Zhang

**Affiliations:** 1 Department of Neurology, Centre for Leading Medicine and Advanced Technologies of IHM, The First Affiliated Hospital of USTC, Division of Life Sciences and Medicine, University of Science and Technology of China, Hefei, Anhui 230001 China; 2 School of Medicine, Anhui University of Science and Technology, Huainan, 232001 Anhui China; 3 School of Basic Medical Sciences, Division of Life Sciences and Medicine, University of Science and Technology of China, Hefei, 230026 Anhui China

**Keywords:** VGLUT3^+^ sensory neuron, NPY^+^ neuron, DYN^+^ neuron, Mechanical itch, Chemical itch, Alloknesis

## Abstract

Itch is a common symptom among patients suffering dermatological and systemic diseases, yet effective clinical treatments are currently lacking. Previous research has suggested that vesicular glutamate transporter 3 (VGLUT3)-lineage sensory neurons may play a role in inhibiting itch, but the circuit mechanisms within the spinal cord remain unclear. In this study, we employed optogenetic techniques to activate VGLUT3-lineage sensory afferents in mice and observed a significant reduction in scratching behaviors elicited by both pruritogens and mechanical stimuli. Moreover, aversive component of chemical itch assessed by conditioned place aversion (CPA) was abrogated. Viral tracing combined with electrophysiological recordings revealed synaptic connections between VGLUT3^+^ sensory neurons and spinal dynorphin (SC^DYN^) /neuropeptide Y-expressing (SC^NPY^) neurons. Further pharmacological studies indicated that intrathecal injection of antagonists of neuropeptide Y1 receptor and kappa opioid receptor (KOR) separately diminished VGLUT3^+^ neurons-mediated inhibitory effects on mechanical and chemical itch, respectively. In summary, our findings suggest that VGLUT3^+^ sensory neurons participate in itch regulation through interactions with two classes of inhibitory neurons in the spinal cord, shedding light on potential therapeutic targets for distinct forms of itch management.

**Supplementary Information:**

The online version contains supplementary material available at 10.1186/s13041-025-01245-3.

## Introduction

Itch is a subjective distressing skin sensation that elicits unpleasant emotions and an urge to scratch. Given its multifactorial etiology and the involvement of multiple neurotransmitters and neural pathways in itch signal transmission, the treatment of itching faces inherent challenges [1–3]. While scratching is a highly effective method for alleviating itch, it can easily lead to an ‘itch-scratch vicious cycle’ [4, 5], ultimately resulting in skin lesions at the itching site and may even progress to ulceration. This paradox has driven investigation into non-lesional itch modulation strategies, with emerging as a potential candidate.

As a sensory organ, the skin expresses a diverse array of receptors, including C-Low-Threshold Mechanoreceptors (C-LTMRs), which are responsible for affective touch transmission [6, 7]. In rodents, C-LTMRs exhibit a distinct gene expression pattern of which *VGLUT3* as one of the markers, distinguishing them from other sensory neurons [8–10]. Mechanistic studies in rodents demonstrate that C-LTMRs mediate touch-evoked itch suppression through spinal inhibition [11], though human psychophysical studies report modest effects [12]. This interspecies discrepancy may reflect either fundamental neurobiological differences or methodological constraints in human itch quantification.

Itch signals are transmitted via sensory fibers to the spinal cord, where interneurons process and relay these signals to ascending projection neurons [3, 13, 14]. Analogous to the gate control theory of pain [15], there exist some types of inhibitory neurons in the spinal dorsal horn, including dynorphin-expressing (DYN^+^) or neuropeptide Y-expressing (NPY^+^) neurons, which act as ‘gatekeepers’ for suppressing the ascending transmission of itch signals. Previous study indicated that DYN^+^ and NPY^+^ neurons are considered a potential therapeutic target for itch regulation. When DYN^+^ neurons are deficient or NPY^+^ neurons are eliminated or silenced, mice exhibit spontaneous scratching behavior, resulting in visibly damaged skin and heightened responses to pruritogens [16–18]. On the contrary, exogenous application of kappa opioid receptor (KOR) agonists can suppress chemical itch [16, 19–21]. Under chronic itch conditions, the reduced activity of inhibitory interneurons in the spinal cord could cause abnormal excitation in the itch circuit [22–24]. Previous research has highlighted that the mechanical itch is governed by the spinal NPY-Y1R-signaling pathway [25, 26]. While a recent study reported that NPY^+^ interneurons are also involved in gating the transmission of chemical itch signals [27], the regulatory effects of NPY^+^ neurons on different types of itch remain controversial.

In this study, we hypothesized that activation of peripheral VGLUT3^+^ nerves would alleviate itch by recruiting spinal cord inhibitory neurons. Using optogenetics to activate peripheral VGLUT3^+^ fibers, we effectively inhibit both chemical itch and mechanical itch/alloknesis, as well as itch-related negative affection. Using a combination of viral tracing, in situ hybridization, electrophysiology, and pharmacology, we proved that spinal NPY-Y1R and DYN-KOR-signaling pathways were crucial for VGLUT3^+^ sensory neurons-mediated mechanical itch/alloknesis and chemical itch inhibition, respectively. Our research reveals the spinal mechanism of touch-mediated itch inhibition and sheds light on potential therapeutic targets for managing different types of itch.

## Results

### Expression profile of channelrhodopsin-2 in VGLUT3-lineage sensory neurons

**Fig. 1:**
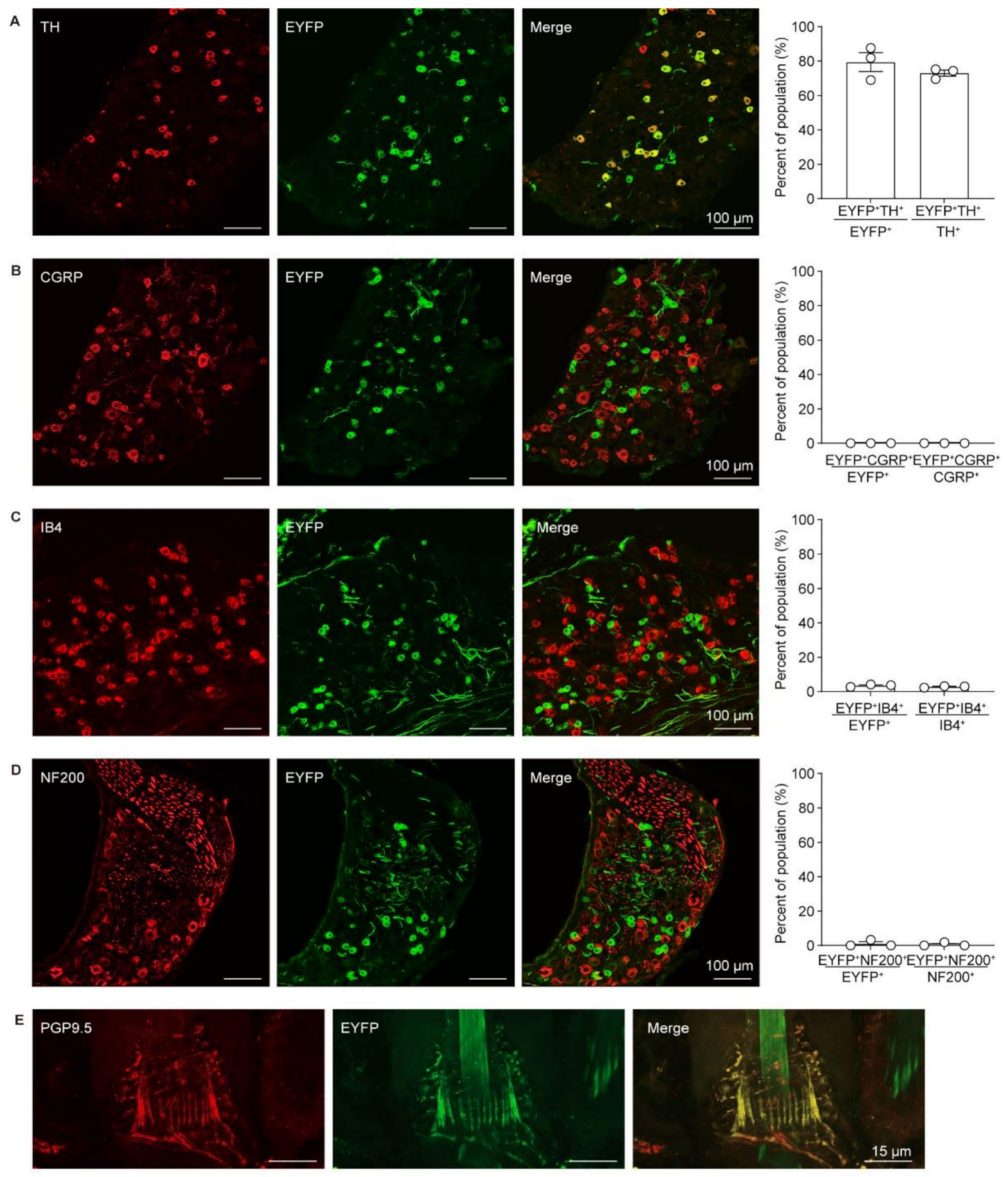
Expression profile of channelrhodopsin-2 in VGLUT3-lineage sensory neurons. **A-D** Representative images (left) of co-localization of ChR2-EYFP with TH^+^ (**A**), CGRP^+^ (**B**), IB4^+^ (**C**), NF200^+^ (**D**) neurons and statistical data (right) in *VGLUT3-ChR2* mice (*n* = 3 mice per group). Scale bar, 100 μm. **E** Representative images demonstrating the co-localization of VGLUT3^+^ fibers with PGP9.5 in hairy nape skin. Scale bar, 15 μm. Data are represented as mean with SEM

To explore the expression profile of VGLUT3-lineage sensory neurons, we generated a heterozygous *VGLUT3*^*cre*^; *ROSA*^*ChR2−EYFP*^ mice (referred to as *VGLUT3-ChR2* mice) by crossing *VGLUT3*^*cre*^ mice with *ROSA*^*ChR2−EYFP*^ mice. The light-activated optogenetic protein channelrhodopsin-2 (ChR2), labeled with EYFP, was selectively expressed in VGLUT3^+^ neurons. To examine the cellular properties of VGLUT3-lineage neurons in the dorsal root ganglia (DRG^VGLUT3^), immunofluorescence of the DRG sections with sensory neuron markers was performed in *VGLUT3-ChR2* mice. We observed that around 81% of DRG^VGLUT3^ neurons co-expressed TH, a marker of C-LTMR (Fig. 1A) [8, 28]. In comparison, none of DRG^VGLUT3^ neurons expressed CGRP^+^ and only few of them expressed IB4^+^ (Fig. 1B C). This is consistent with previous report that DRG^VGLUT3^ neurons do not belong to either peptidergic or non-peptidergic neurons subtypes [29]. Moreover, NF200, a marker indicative of large-diameter myelinated A-fibers [30, 31], was nearly absent in DRG^VGLUT3^ neurons (Fig. 1D). From above, VGLUT3-lineage sensory neurons represent a subpopulation of unmyelinated C-LTMRs. To assess the viability of optogenetic manipulation of VGLUT3^+^ sensory fibers through transdermal illumination, we examined the expression of VGLUT3^+^ nerve terminals in the hairy nape skin. We detected dense ChR2-EYFP signals in the nape skin which were co-localized with the nerve fiber marker PGP9.5 [32] (Fig. 1E).

### Activation of VGLUT3^+^ sensory nerves relieves both sensory and affective dimensions of itch

**Fig. 2:**
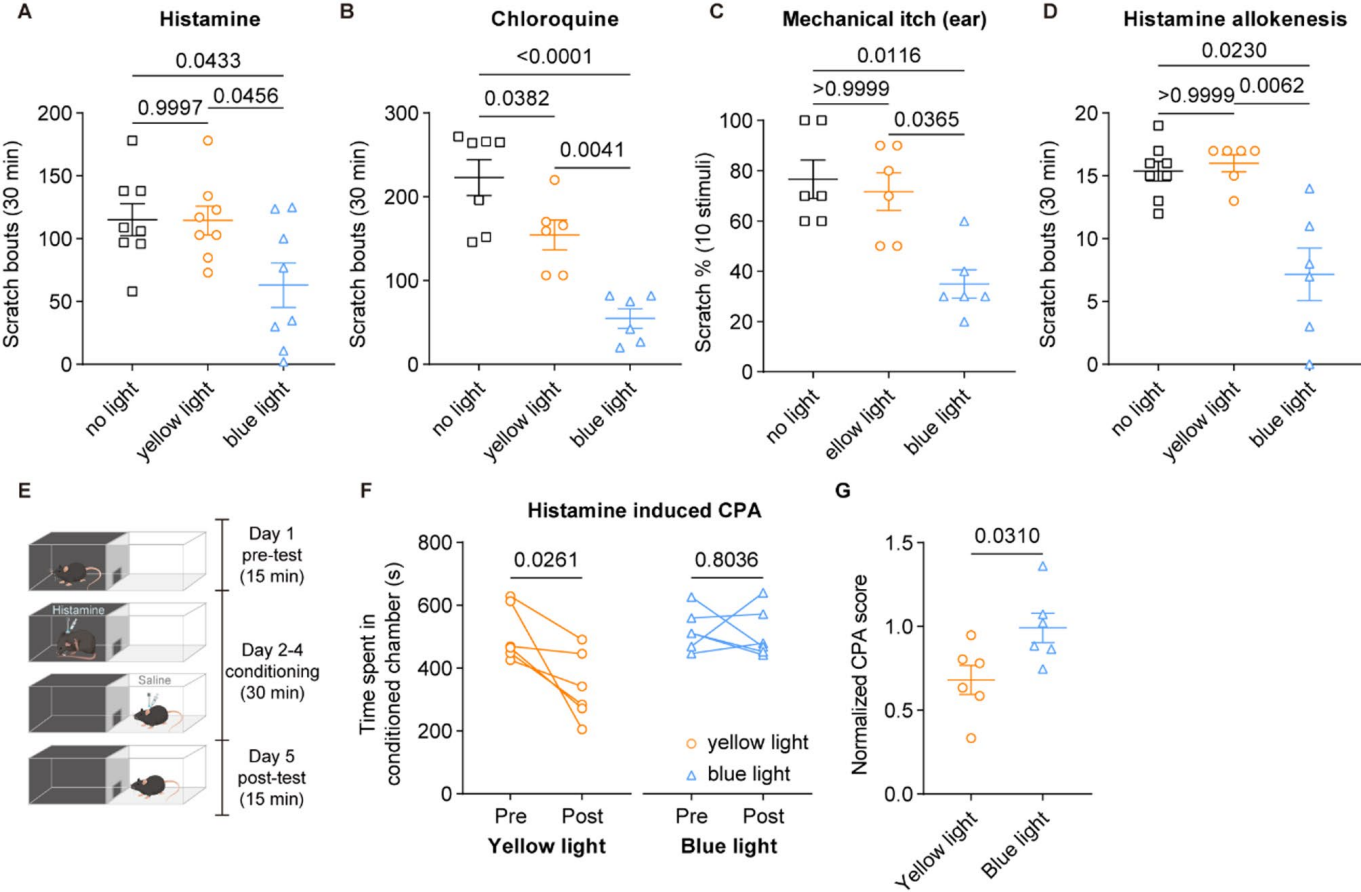
Activation of VGLUT3-lineage sensory nerves relieves both sensory and affective dimensions of itch. **A**,** B** Scratching responses assessed for 30 min following histamine (**A**) or chloroquine (**B**) injection, with concurrent yellow or blue light exposure, or no light treatment. **C** Measurement of acute mechanical itch, with concurrent yellow or blue light exposure, or no light treatment. **D** Alloknesis score assessed for 30 min following 0.07 g von Frey filament induction, with concurrent yellow or blue light exposure, or no light treatment. **E** Schematics of itch-evoked CPA apparatus and experimental design. **F**,** G** Loss of histamine-evoked aversion following activation of VGLUT3^+^ fibers. Statistic data showing time spent in conditioned chamber (**F**) and the normalized CPA score (**G**). Data are represented as mean with SEM and assessed using one-way ANOVA, two-tailed paired or unpaired *t* test, exact *p*-values are reported for all statistical comparisons

Itch can be evoked by various stimuli, including innocuous mechanical stimuli on the skin, referred to as mechanical itch [33], or chemical mediators, referred to as chemical itch [34]. To investigate the role of VGLUT3-lineage neurons in acute itch regulation, we quantified scratch bouts in *VGLUT3-ChR2* mice using various itch models. First, two types of acute chemical itch, histamine-dependent and non-histaminergic itch [1], were examined. The mice had their nape skin shaved and then exposed to yellow or blue light exposure, or no light treatment instantly after intradermal injection of histamine or chloroquine. We found that activation of peripheral VGLUT3-lineage sensory nerves markedly reduced the number of scratch bouts in mice (histamine: no light vs. yellow light, *p* = 0.9997, no light vs. blue light, *p* = 0.0433, yellow light vs. blue light, *p* = 0.0456, one-way ANOVA; chloroquine: no light vs. yellow light, *p* = 0.0382, no light vs. blue light, *p* < 0.0001, yellow light vs. blue light, p=0.0041, one-way ANOVA; Fig. 2A and B). In addition to acute chemical itch, we also examined whether the same manipulation had an impact on acute mechanical itch. As reported previously [26], light punctate stimuli (0.07 g von Frey filament) poking the shaved skin behind the ear in naïve mice elicited hindpaw scratching bouts directed toward the stimulus (no light vs. yellow light, *p* > 0.9999, no light vs. blue light, *p* = 0.0116, yellow light vs. blue light, *p* = 0.0365, one-way ANOVA) (Fig. 2C). However, when VGLUT3-lineage sensory fibers behind the ear were concurrently activated, the observed acute mechanical itch was alleviated. Mechanical itch sensitization is one of hallmarks of chronic itch [35–37]. In healthy humans and rodents [38, 39], intradermal injection of histamine provokes mechanical alloknesis in which lightly touching normal skin near the injection site evokes nocuous itch sensation. We then assessed the role of DRG^VGLUT3^ neurons in restoring mechanical itch sensitization in the mouse model of histamine-induced alloknesis. In this experiment, *VGLUT3-ChR2* mice were intradermally injected with a low dose of histamine in the nape. Vehicle-injected *VGLUT3-ChR2* mice were used as control. After 30 min, mice were poked with a 0.07 g von Frey filament surrounding the injection site. In control *VGLUT3-ChR2* mice, light punctate stimuli on the nape evoked rare scratching responses, while the same stimuli evoked reliable responses in histamine-injected *VGLUT3-ChR2* mice. However, when VGLUT3-lineage fibers were activated by blue light, the alloknesis was significantly alleviated compared to yellow or no light group (no light vs. yellow light, *p* > 0.9999, no light vs. blue light, *p* = 0.0230, yellow light vs. blue light, *p* = 0.0062, one-way ANOVA) (Fig. 2D). These results suggest that histamine-induced mechanical alloknesis, which mimics central sensitization under chronic itch conditions [40], was also inhibited by VGLUT3-lineage sensory neurons. 

Aversion is a common indicator of the emotional dimension of itch [41]. To evaluate the role of VGLUT3^+^ sensory neurons in regulating itch-related aversive behavior, we performed a conditioned place aversion (CPA) test on *VGLUT3-ChR2* mice (Fig. 2E). During three consecutive days-conditioning by histamine injection, mice were simultaneously subjected to the yellow light or blue light stimulation of the injection site. We observed that mice with yellow light stimulation spent less time in histamine-paired chamber during the post-training test when compared to the pre-training test. (pre-test: 508.3 ± 36.26s, post-test: 340.0 ± 44.73s; t = 3.124, *p* = 0.0261, two-tailed paired t test) (Fig. 2F, left). In contrast, blue light-stimulated mice spent comparable time in histamine-paired chamber (pre-test: 521.0 ± 26.48s, post-test: 509.0 ± 32.35s; t = 0.2623, *p* = 0.8036, two-tailed paired *t* test) (Fig. 2F, right). Correspondingly, the aversion score was only observed decreased in yellow light group, indicating an evident aversion to the itch-paired chamber, but not blue light-stimulated mice (yellow light vs. blue light, t = 2.508, *p* = 0.0310, two-tailed unpaired *t* test) (Fig. 2G). Altogether, our data suggest that VGLUT3-lineage sensory neurons regulate both sensory and emotional components of itch.

### VGLUT3^+^ sensory neurons target spinal DYN and NPY-expressing neurons

**Fig. 3:**
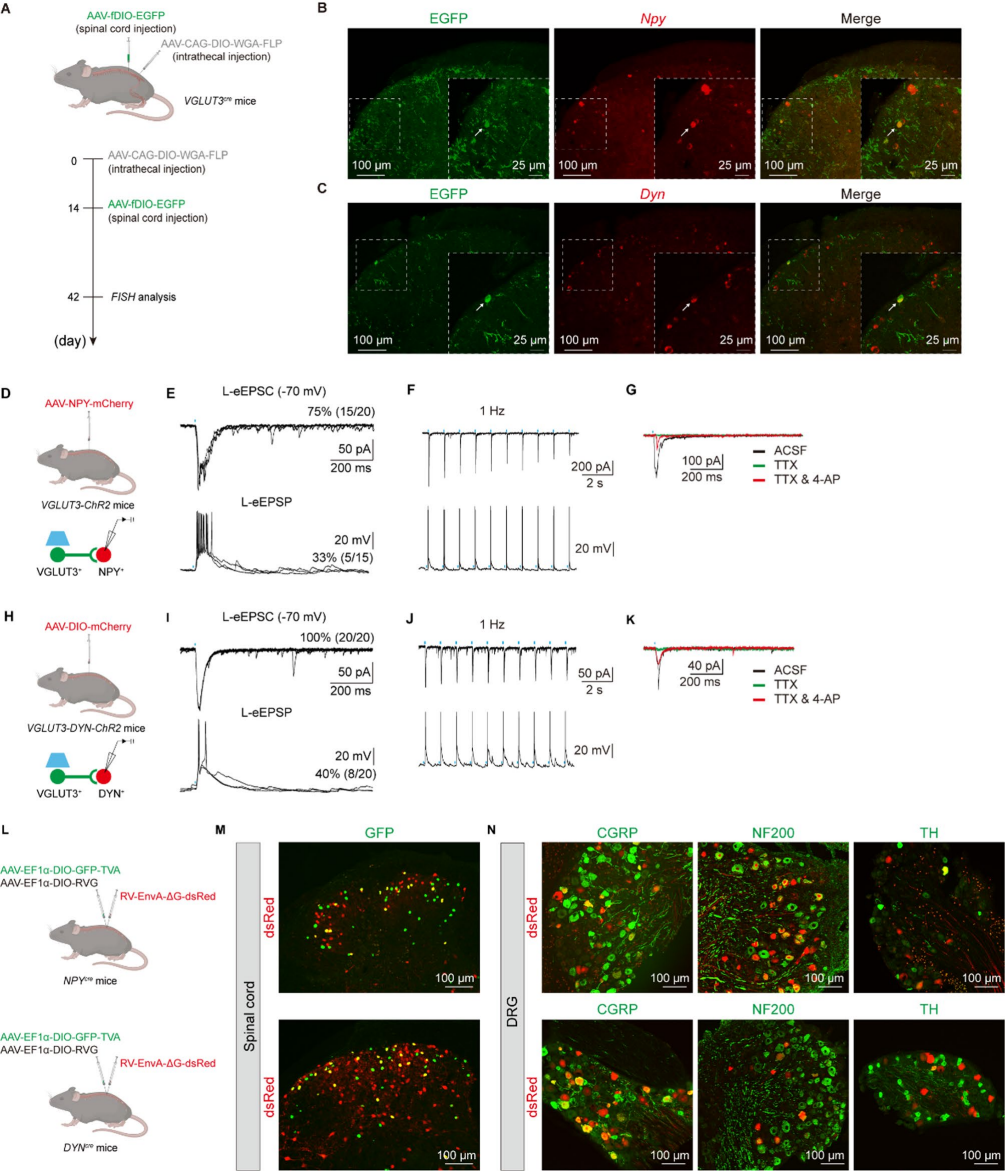
VGLUT3^+^ sensory neurons target spinal DYN and NPY-expressing neurons. **A** Schematic of AAV-CAG-DIO-WGA-FLP intrathecal injection and AAV-fDIO-EGFP intraspinal injection in the L3-L5 segments in *VGLUT3*^*Cre*^ mice. **B**,** C** Representative images showing co-localization of EGFP with *Npy* (**B**) or *Dyn* (**C**) mRNA (*FISH*, red) by fluorescence in situ hybridization. Arrows indicate double-positive cells for indicated mRNA and EGFP. Scale bar, 100 μm. Bottom panels are higher magnification from their respective insets. Scale bar, 25 μm. **D**,** H** Schematic of intraspinal virus injection in the L3-L5 segments (top) and recorded neurons (bottom). **E**,** I** Representative traces of L-eEPSCs (top) and action potentials (bottom) in the NPY-mCherry^+^ neurons (**E**) and DYN-mCherry^+^ neurons (**I**). **F**,** J** L-eEPSCs (top) and APs (bottom) in the NPY-mCherry^+^ neuron (**F**) and the DYN-mCherry^+^ neuron (**J**) following 1 Hz light stimulation of DRG^VGLUT3^. **G**,** K** Representative traces of L-eEPSCs in mCherry^+^ neurons before (ACSF) and after TTX (1 µM), TTX&4-AP (300 µM) treatment. **L** Schematic of retrograde tracing in *NPY*^*Cre*^ (top) and *DYN*^*Cre*^ (bottom) mice. **M** Sections of spinal cord dorsal horn from *NPY*^*Cre*^ (top) and *DYN*^*Cre*^ (bottom) mice injected with AAV helper viruses (green) and rabies virus (RVG, red). Scale bar, 100 μm. **N** Representative images of co-localization of dsRed with GCRP^+^ (left), NF200^+^ (middle), TH^+^ (right) neurons in the DRG in *NPY*^*Cre*^ (top) and *DYN*^*Cre*^ (bottom) mice. Arrows indicate double-positive cells for TH and dsRed. Scale bar, 100 μm

Recent research has been dedicated to exploring the transmission and procession of itch information within the spinal cord [42–44]. Several studies have indicated that the activation of spinal inhibitory interneurons might effectively dampen pruritus transmission [16–18, 20, 26]. Given the itch inhibition following VGLUT3-lineage sensory nerve activation, we proposed that DRG^VGLUT3^ neurons could activate spinal inhibitory interneurons that ‘gate’ itch transmission. To validate this hypothesis, we traced DRG^VGLUT3^-downstream spinal neurons. To this end, we performed intrathecal injection of a cre-dependent AAV-expressing the anterograde trans-synaptic tracer wheat germ agglutinin (AAV-CAG-DIO-WGA-Flp) in adult *VGLUT3*^*cre*^ mice, enabling the selective expression of WGA-Flp in DRG^VGLUT3^ neurons. Two weeks later, we injected a FlpO-dependent AAV-fDIO-EGFP virus into the spinal cord [45, 46]. Through this strategy, we successfully achieved anterograde trans-synaptic labeling of DRG^VGLUT3^-downstream spinal neurons that express EGFP (Fig. 3A). To characterize the identity of EGFP^+^ spinal cord neurons, we subsequently investigated their co-localization with DYN and NPY-expressing spinal cord neurons (SC^DYN^/SC^NPY^) which have been previously implicated in the inhibition of pruritogen-responsive neurons [16, 18]. Fluorescence in situ hybridization results showed that some of EGFP^+^ neurons were co-localized with *NPY-RNA* or *DYN-RNA* probe (Fig. 3B and C), suggesting the presence of DRG^VGLUT3^→SC^NPY^ and DRG^VGLUT3^→SC^DYN^ pathway.

To further examine the functional connection of DRG^VGLUT3^→SC^NPY^ pathway, the AAV-NPY-mCherry virus, was injected into the spinal cord to label NPY^+^ neurons. To validate the virus specificity, we concurrently injected AAV-DIO-EYFP into the dorsal horn of *NPY*^*cre*^ mice (Fig. S1A). After 3 weeks, we observed high co-localization of mCherry and EYFP signals in the dorsal horn (Fig. S1B, C), proving the reliability of AAV-NPY-mCherry to label NPY^+^ cells. Thus, we injected this virus into the spinal cord of *VGLUT3-ChR2* mice to assess the postsynaptic responses of SC^NPY^ neurons following DRG^VGLUT3^ cell excitation (Fig. 3D). DRG-dorsal root-attached spinal slices were then prepared as previously [47, 48]. Whole-cell patch clamp recordings were conducted on SC^NPY^ neurons and the blue light was illuminated to the DRG. Among the twenty mCherry-labeled neurons recorded, fifteen neurons exhibited light-evoked excitatory postsynaptic currents (L-eEPSCs). Notably, one third of them fired light-evoked action potentials (L-eAPs) (Fig. 3E). Moreover, all neurons (9 of 9) that displayed L-eEPSCs/eAPs demonstrated a lack of failures following 1 Hz light stimulation (Fig. 3F), further support the monosynaptic connections. The L-eEPSCs could be blocked by TTX (1 µM) application and partially rescued by potassium channel blocker 4-aminopyridine (4-AP, 300 µM) (Fig. 3G). Thus, spinal NPY^+^ neurons receive monosynaptic innervation from VGLUT3-expressing DRG neurons.

To further investigate the monosynaptic connections of the DRG^VGLUT3^ fibers innervating SC^DYN^ neurons, we employed a similar strategy. The AAV-DIO-mCherry virus was injected into the spinal cord of *VGLUT3-DYN-ChR2* mice to label DYN^+^ neurons (Fig. 3H). To confirm the specificity of the viral labeling, we injected AAV-DIO-EYFP into *DYN-Ai14* mice (Fig. S1D). High co-localization of tdTomato^+^ and EYFP^+^ signals was observed in the dorsal horn (Fig. S1 E, G), validating the reliability of AAV-DIO-mCherry for labeling DYN^+^ cells. Whole-cell patch clamp recordings were conducted on SC^DYN^ neurons and the blue light was illuminated to the DRG. 100% (20/20) SC^DYN^ neurons had detectable L-eEPSCs, and 40% (8/20) of them fired APs (Fig. 3I). Furthermore, no failures were observed following 1 Hz light stimulation and recovery of L-eEPSCs following TTX and 4-AP treatment (Fig. 3J, K), indicate the monosynaptic connections of DRG^VGLUT3^→SC^DYN^ pathway.

To further confirm the monosynaptic connections from DRG^VGLUT3^→SC^DYN^ and DRG^VGLUT3^→SC^NPY^ pathway, we performed rabies virus-mediated retrograde trans-synaptic tracing combined with immunofluorescence staining (Fig. 3L-N). The helper virus into the spinal cord of *DYN*^*cre*^ mice or *NPY*^*cre*^ mice. Two weeks later, the rabies virus RV-EnvA-ΔG-dsRed was injected into same site in the spinal cord. Retrograde dsRed expression was observed in several cutaneous subtypes, including CGRP^+^-nociceptors, NF200^+^-Aβ-LTMRs, and TH^+^-C-LTMRs. In summary, this analysis provides clear evidence that the SC^DYN^ and SC^NPY^ neurons are extensively innervated by DRG^VGLUT3^ fibers.

### Chemogenetic activation of DYN^+^ and NPY^+^ spinal neurons suppresses chemical and mechanical itch, respectively

**Fig. 4:**
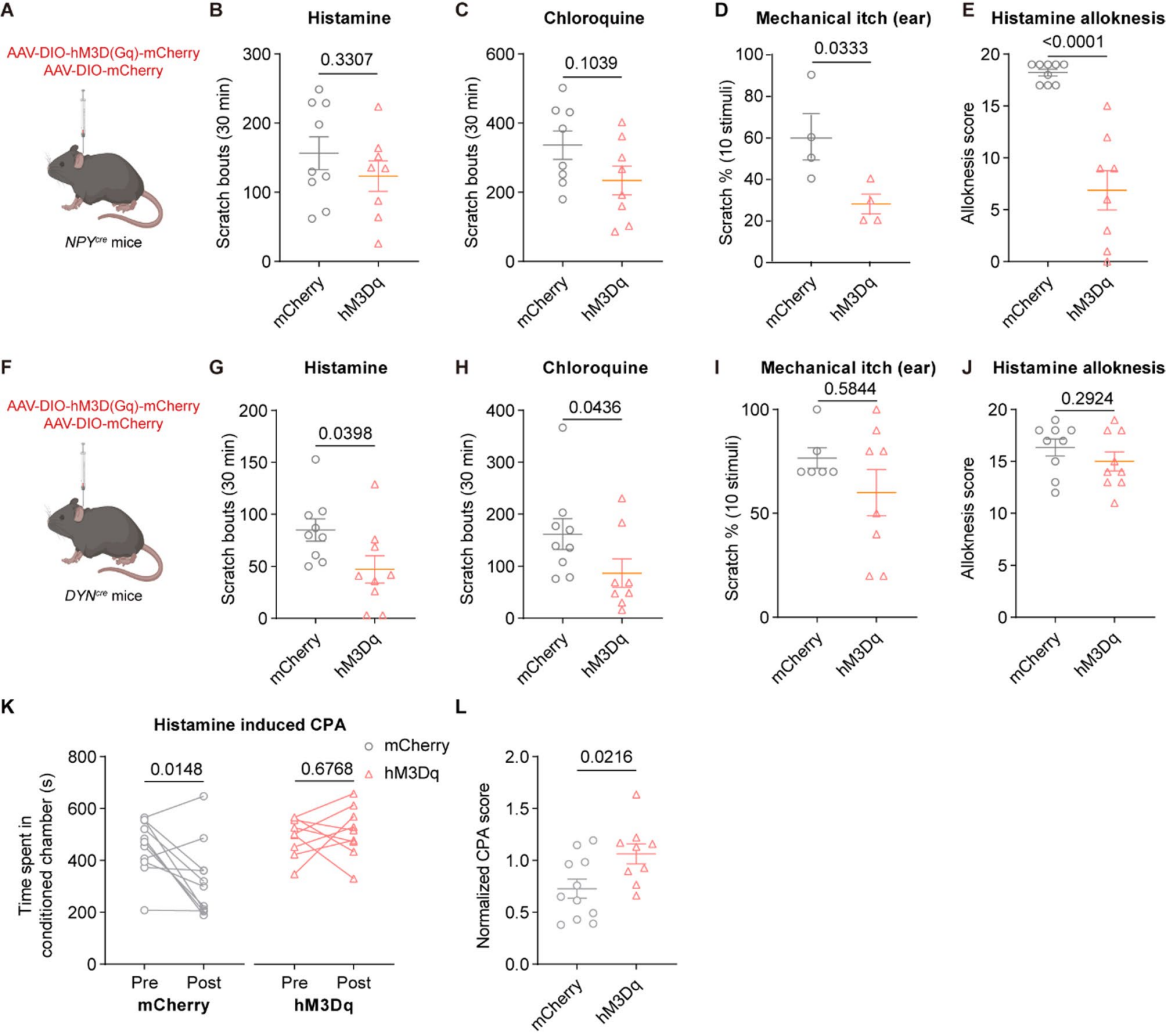
Chemogenetic activation of DYN^+^ and NPY^+^ spinal neurons suppresses chemical and mechanical itch, respectively. **A** Schematic of hM3Dq intraspinal injection in the thoracic level in *NPY*^*Cre*^ mice. **B**,** C** Scratching responses assessed for 30 min following histamine (**B**) or chloroquine (**C**) injection, with concurrent activation of NPY^+^ neurons by chemogenetics. **D** Measurement of acute mechanical itch, with activation of NPY^+^ neurons by chemogenetics. **E** Alloknesis score assessed for 30 min following 0.07 g von Frey filament induction, with concurrent activation of NPY^+^ neurons by chemogenetics. **F** Schematic of hM3Dq intraspinal injection in the thoracic level in *DYN*^*Cre*^ mice. **G**,** H** Scratching responses assessed for 30 min following histamine (**G**) or chloroquine (**H**) injection, with concurrent activation of DYN^+^ neurons by chemogenetics. **I** Measurement of acute mechanical itch, with concurrent activation of DYN^+^ neurons by chemogenetics. **J** Alloknesis score assessed for 30 min following 0.07 g von Frey filament induction, with concurrent activation of DYN^+^ neurons by chemogenetics. **K**,** L** Statistic data showing blockage of histamine-evoked aversion following activation of DYN^+^ spinal neurons. Time spent in conditioned chamber (s) (**K**) and the normalized CPA score (**L**). Data are represented as mean with SEM and assessed using two-tailed paired or unpaired *t* test, exact *p*-values are reported for all statistical comparisons

SC^NPY^ neurons have been widely considered to be involved in the specific modulation of mechanical itch [18, 25, 26]. However, anti-chemical itch effects [27, 49, 50] were also reported. To investigate the exact role of adult SC^NPY^ neurons in itch regulation, we examined the impact of hM3Dq-mediated activation of these neurons on itch behaviors. To this end, AAV-DIO-hM3Dq-mCherry or a control virus was injected into the cervical (C3–C5) spinal cord of *NPY*^*cre*^ mice (Fig. 4A). SC^NPY^ neural activation was achieved through intraperitoneal CNO injection. In mCherry group, both histamine and chloroquine injections provoked robust scratching responses. However, hM3Dq-injected mice had the comparable number of scratching responses (histamine: t = 1.005, *p* = 0.3307, two-tailed unpaired *t* test; chloroquine: t = 1.739, *p* = 0.1039, two-tailed unpaired *t* test) (Fig. 4B and C), indicating that spinal NPY^+^ neuron activities do not affect chemical itch. We further examined mechanical itch sensitivity in a naïve condition and the histamine-induced alloknesis model. Light punctate stimuli applied to shaved skin behind the ears or on the nape elicited scratching behavior towards the stimulus area in the NPY-mCherry group. However, activation of SC^NPY^ neurons significantly attenuated touch-evoked scratching (mechanical itch: t = 2.751, *p* = 0.0333, two-tailed unpaired *t* test; histamine-induced alloknesis: *p* < 0.0001, Mann Whitney test) (Fig. 4D, E). Taken together, adult SC^NPY^ are preferentially involved in gating mechanical itch.

SC^DYN^ neurons have been demonstrated to exert critical inhibitory effects on chemical itch transmission through GABAergic modulation of GRPR^+^ neurons in the spinal cord [2, 16]. We applied a similar approach to specifically activate SC^DYN^ neurons and conducted the same behavioral assays as described above (Fig. 4F). Contrary to SC^NPY^ neurons, chemogenetic activation of SC^DYN^ neurons significantly alleviated histamine or chloroquine-induced chemical itch (histamine: t = 2.237, *p* = 0.0398, two-tailed unpaired *t* test; chloroquine: *p* = 0.0436, Mann Whitney test) (Fig. 4G and H), rather than affecting mechanical itch in a naive condition and the histamine-induced alloknesis model (mechanical itch: *p* = 0.5844, Mann Whitney test; histamine-induced alloknesis: t = 1.089, *p* = 0.2924, two-tailed unpaired *t* test) (Fig. 4I-J). Next, we conducted the CPA test to assess the contribution of SC^DYN^ neurons to histamine-induced aversive behavior. After receiving histamine injections for three consecutive days, mCherry control mice spent significantly less time in the histamine-paired black compartment, indicating an evident aversion to the itch-paired chamber. In contrast, hM3Dq mice spent similar time in two compartments and didn’t show a significant aversive response (mCherry: pre-test: 454.1 ± 31.78s, post-test: 319.3 ± 43.01s; t = 2.938, *p* = 0.0148, two-tailed paired *t* test; hM3Dq: pre-test: 493.3 ± 24.62s, post-test: 511.4 ± 32.70s; t = 0.4325, *p* = 0.6768, two-tailed paired *t* test; CPA score: t = 2.515, *p* = 0.0216, two-tailed unpaired *t* test) (Fig. 4K, L). In summary, our results suggest that adult SC^NPY^ neurons selectively modulates mechanical itch, while adult SC^DYN^ neurons preferentially regulates sensory and affective components of chemical itch.

### VGLUT3^+^ sensory fibers alleviate mechanical and chemical itch via spinal NPY-Y1R and DYN-KOR-signaling pathway, respectively

**Fig. 5:**
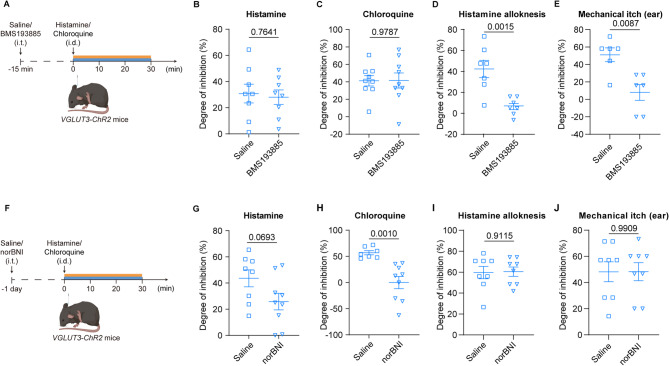
VGLUT3^+^ sensory fibers alleviate mechanical and chemical itch via spinal NPY-Y1R and DYN-KOR-signaling pathway, respectively. **A, F** Schematic of experimental design. **B, C** Pretreatment of Y1 receptor antagonist (BMS193885) does not alter the inhibitory effect of VGLUT3^+^ fibers on both histamine and chloroquine itch models. **D, E** Pretreatment of BMS193885 abrogates the inhibitory effect of VGLUT3^+^ fibers on mechanical itch and reduces the degree of inhibition. **G, H **Pretreatment of KOR antagonist (norBNI) attenuates the degree of itch inhibition mediated by VGLUT3^+^ fibers on both histamine and chloroquine itch models. **I,J** Pretreatment of KOR antagonist (norBNI) does not alter VGLUT3^+^ fiber-mediated suppression of mechanical itch. Data are represented as mean with SEM and assessed using two-tailed unpaired* t* test, exact p-values are reported for all statistical comparisons

Given that spinal NPY^+^ and DYN^+^ neurons suppress itch via NPY-Y1R and dynorphin-KOR-signaling pathway, respectively [16, 25, 27, 51–53], we next investigate whether these two pathways play essential roles in itch inhibition by VGLUT3^+^ sensory fibers. First, we assessed the necessity of the NPY-Y1R-signaling pathway in DRG^VGLUT3^-mediated itch inhibition. We intrathecally injected BMS193885, a specific Y1R antagonist, in *VGLUT3-ChR2* mice. Fifteen minutes later, histamine or chloroquine was injected into the nape skin and VGLUT3^+^ fibers were simultaneously stimulated by blue light (or yellow light as a control) (Fig. 5A). The scratching bouts within 30 min were quantified. In saline-treated mice, scratching responses induced by either histamine were all significantly reduced in blue light group when compared to the yellow light group. In BMS193885-treated mice, the itch inhibition by VGLUT3^+^ fibers was still observed in both histamine and chloroquine itch models. We further compared the extent of itch inhibition by VGLUT3^+^ fibers between BMS193885-treated mice and saline-treated mice. Indeed, in the chemical itch model, blocking the NPY-Y1R-signaling pathway does not diminish the itch inhibition mediated by VGLUT3^+^ sensory fibers (histamine: t = 0.3059, *p* = 0.7641, two-tailed unpaired *t* test; chloroquine: t = 0.02711, *p* = 0.9787, two-tailed unpaired *t* test) (Fig. 5B, C). We further explored the necessity of the NPY-Y1R-signaling pathway in DRG^VGLUT3^-mediated inhibition of mechanical itch. Interestingly, the inhibitory effect of VGLUT3^+^ fibers on mechanical itch was almost abrogated by pretreatment with BMS193885 (histamine alloknesis: t = 4.080, *p* = 0.0015, two-tailed unpaired *t* test; mechanical itch: *p* = 0.0084, Mann Whitney test) (Fig. 5D, E). These data revealed that VGLUT3^+^ fibers inhibit mechanical itch by recruiting NPY-Y1R-signaling pathway.

Finally, we assessed the necessity of the DYN-KOR-signaling pathway in itch-inhibitory effects of VGLUT3^+^ sensory fibers (Fig. 5F). In consistent with above results, in saline-treated mice, blue light activation of VGLUT3^+^ fibers greatly reduced scratching responses induced by both histamine and chloroquine. In norBNI-pretreated mice, while the itch inhibition still remained, the degree of itch inhibition had strong tendency to be reduced in histamine model (t = 1.956, *p* = 0.0693, two-tailed unpaired *t* test) (Fig. 5G) and was significantly attenuated in chloroquine model (t = 4.116, *p* = 0.0010, two-tailed unpaired *t* test) (Fig. 5H). These results revealed that VGLUT3^+^ fibers inhibit chemical itch by recruiting DYN-KOR-signaling pathway. To determine pathway specificity, we extended this analysis to mechanical itch paradigms. In the histamine alloknesis model, norBNI pretreatment did not alter VGLUT3⁺ fiber-mediated suppression of von Frey-evoked scratching (t = 0.1132, *p* = 0.9115; Fig. 5I). Similarly, in the mechanical itch model, optogenetic activation reduced scratching by 48.21 ± 7.606% in vehicle controls versus 48.33 ± 6.872% with norBNI (t = 0.01161, *p* = 0.9909; Fig. 5J), demonstrating that KOR antagonism does not compromise mechanical itch inhibition. Collectively, DRG^VGLUT3^ neurons inhibit both mechanical and chemical itch through two parallel pathways involving spinal NPY^+^ and DYN^+^ interneurons, respectively.

## Discussion

**Fig. 6:**
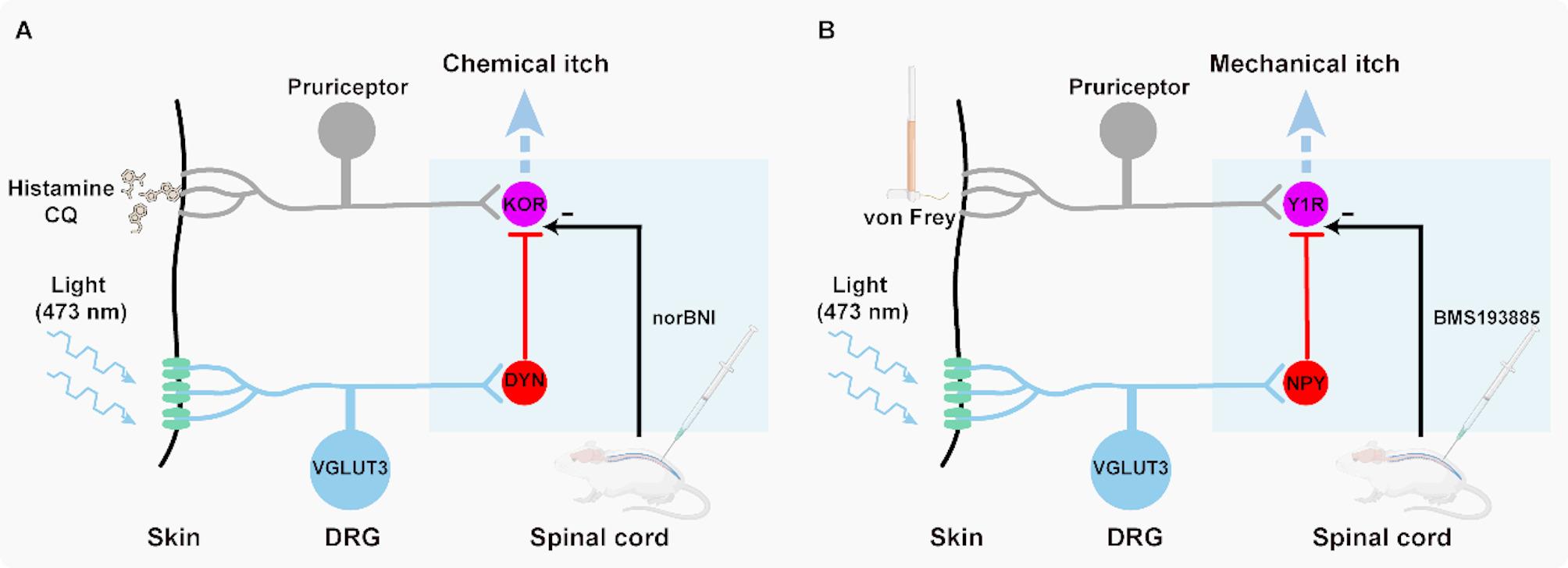
Schematic model of the spinal inhibitory circuit for DRG^VGLUT3^-mediated suppression of itch. **A** Optogenetic activation of VGLUT3-expressing LTMR terminals inhibits itch signaling in the spinal cord through distinct pathways. Chemical itch is suppressed via a dynorphin (DYN)-mediated inhibitory pathway, where VGLUT3⁺ LTMR activation recruits DYN⁺ inhibitory interneurons to suppress κ-opioid receptor (KOR)-expressing pruriceptive neurons. This anti-pruritic effect is abolished by the selective KOR antagonist norBNI. **B** Mechanical itch is inhibited through a distinct neuropeptide Y (NPY)-dependent pathway, where VGLUT3⁺ LTMR activation recruits NPY⁺ inhibitory interneurons to suppress itch-transmission neurons via Y1 receptor (Y1R) signaling. This anti-pruritic effect is selectively blocked by the Y1R antagonist BMS193885

The present work shows that optogenetic activation of VGLUT3^+^ nerve terminals inhibited scratching behavior in mice induced by both chemical and mechanical itch. By using viral tracing, in situ hybridization and electrophysiological verification, we identified synaptic connections between DRG^VGLUT3^ neurons and SC^DYN^/SC^NPY^ neurons that gate itch. Blocking spinal NPY-Y1R and DYN-KOR- signaling pathway disrupted the inhibitory effects of VGLUT3^+^ neurons on chemical and mechanical itch, respectively (Fig. 6). These findings offer the opportunity to develop specific targets for treating distinct forms of itch.

### Antipruritic effects of C-LTMRs

C-LTMRs have been extensively studied in animal research [54, 55]. These receptors demonstrate a unique response to tactile stimuli, with the highest firing rates occurring when the friction velocity falls within the range of 1–10 cm/s [6]. This response pattern follows a velocity-dependent inverted U-shaped distribution, which is associated with pleasurable tactile stimuli [6, 56–59]. In vivo single-unit recording from the lumbar spinal cord showed that stroking reduced the responses of spinal neurons to pruritogens [11]. Conversely, aged *HomNa*_*v*_*1.7*^*I228M*^ mice, whose C-LTMR-associated gene expression is profoundly decreased, display skin lesions on the facial and dorsal skin [60]. The unmyelinated C-LTMRs has been characterized due to their distinctive expression profile, including TH, VGLUT3, TAFA4 and GINIP [28, 61, 62]. Previous studies suggested that menthol can inhibit itch on the skin or mucous membranes [63, 64], and the sensitivity to menthol was restricted to the VGLUT3^+^ population [65]. Therefore, VGLUT3^+^ C-LTMRs could serve as a potential target for relieving itch. Ginty and colleagues recently demonstrated that optogenetic activation of TH^+^ neurons (encompassing C-LTMRs) elicits wet dog shake (WDS) behavior in mice [66], which may play a role in remove irritants or potential threats. However, optogenetic activation of VGLUT3⁺ neurons in our *VGLUT3-ChR2* mice failed to induce WDS. A possible explanation lies in the incomplete overlap between these populations: only ~ 80% of TH⁺ neurons co-express VGLUT3 [8]. The remaining 20% of TH⁺/VGLUT3⁻ neurons may constitute a functionally distinct subpopulation responsible for WDS. Another possible reason is that in our experiment, mice were injected with histamine/ chloroquine, which may have affected the occurrence of WDS behavior.

Our findings are consistent with previous observations that specific activation of VGLUT3^+^ nerve terminals inhibits histamine and chloroquine-induced chemical itch [11]. In addition, we revealed that VGLUT3^+^ fibers activation alleviates itch-related negative emotions. Importantly, mechanical alloknesis, frequently occurs in dry skin-based disorders such as psoriasis and atopic dermatitis, is found to be relieved by VGLUT3^+^ fibers excitation (Fig. 1). We thus provide some perspectives on the understanding of VGLUT3^+^ sensory neurons in itch modulation. Itch perception, particularly its affective dimension involving unpleasantness and the urge to scratch, is ultimately processed and integrated within higher brain centers. Recent studies highlight the critical role of cortical and subcortical structures in encoding itch sensation and its associated negative affect [4, 67, 68]. For instance, Ko et al. identified modality-specific neurons within the anterior cingulate cortex (ACC) that are selectively activated by pruritogens rather than pain stimuli [68]. While our study delineates a spinal circuit mechanism (DRG^VGLUT3^→SC^DYN/NPY^ interneurons) underlying the inhibition of itch signaling at the spinal level, it is plausible that dampening spinal itch transmission through this pathway also reduces the ascending drive to cortical itch-processing centers like the ACC. Consequently, the attenuation of itch-related aversion observed in our study (Fig. 2) may reflect not only reduced spinal signaling but also diminished cortical processing of itch unpleasantness mediated by ACC neurons.

### Investigation of downstream spinal neurons targeted by DRG^VGLUT3^ neurons

VGLUT3-expressing LTMRs has been reported to terminate within II–III layers of the spinal cord, establishing synaptic connections with various populations of excitatory and inhibitory interneurons in the dorsal horn [69]. This observation was corroborated in a study involving *VGLUT3-ChR2* mice, in which optogenetic activation of DRG^VGLUT3^ neurons elicited a notable EPSC response in 89% of spinal neurons [70]. Employing a similar electrophysiological approach in *VGLUT3-ChR2* mice, we recorded two distinct categories of light-evoked synaptic responses. In consistent with the study from Honsek et al. [70], our electrophysiological recording found a subset of neurons (27%, 8/30) displayed pure EPSC. Notably, we also observed another subpopulation of neurons (33%, 10/30) with both EPSC and IPSC (Fig. S2). This finding suggests that the activation of VGLUT3^+^ sensory neurons must recruit inhibitory neurons within the spinal cord. Indeed, when we recorded SC^NPY^ and SC^DYN^ neurons, we found most of them had detectable L-eEPSCs and some of cells fired APs.

Due to the low viral infection efficiency of DRG neurons, our anterograde tracing data showed few EGFP^+^ neurons in the spinal cord (Fig. 3). Therefore, we also make an endeavor by using rabies-mediated retrograde tracing from SC^DYN^ and SC^NPY^ neurons, but we did not obtain effective infection in DRG^VGLUT3^ neurons (Fig. 3L-N). This may result from the viral resistance of TH^+^ neurons [18, 23, 71–74]. Future electrophysiology combined with single-cell RT-PCR can be used to identify the connection between sensory neurons and interneurons in the spinal cord [75].

### DRG^VGLUT3^ neurons inhibits itch through circuits involving NPY-Y1R and DYN-KOR-signaling pathway

DYN^+^ and NPY^+^ neurons represent distinct non-overlapping subpopulations of inhibitory neurons in the spinal cord [76] and regulate pain and itch sensation [13, 77]. In the development of DYN^+^ inhibitory neurons, Bhlhb5 play an essential role, and the mice lacking Bhlhb5 neurons exhibit widespread licking, biting, and spontaneous scratching behavior, which remains unalleviated even with the application of menthol [16, 17]. Consistently, the use of KOR agonists has become a therapeutic approach for certain types of chronic itch [78]. In agreement with these findings, we found that chemogenetic activation of persistent SC^DYN^ neurons relieved pruritogen-evoked chemical itch and aversion (Fig. 4). Therefore, in the spinal cord, DYN^+^ inhibitory neurons are pivotal components of the anti-itch pathway [79]. An electrophysiological investigation has demonstrated that throughout postnatal development, DYN^+^ interneurons consistently receive input from low-threshold C-fibers [80]. Furthermore, stimulation of Aδ-fibers and C-LTMR fibers can induce the release of dynorphin, subsequently activating kappa opioid receptors [81], suggesting the potential existence of synaptic connections between DRG^VGLUT3^ neurons and SC^DYN^ neurons. We supported this point by anterograde virus tracing and *FISH* data (Fig. 3). Importantly, we demonstrated that DRG^VGLUT3^ neurons-mediated inhibition of chemical itch was attenuated by KOR antagonists (Fig. 5). Thus, we provide one of spinal circuit substrates for touch inhibition of chemical itch.

NPY^+^ neurons have been extensively reported to gate mechanical itch, but their role in chemical itch remains controversial [18, 25, 27, 49, 50]. On the one hand, Bourane et al. found that ablating *NPY*^*cre*^ lineage neurons in the spinal dorsal horn induced spontaneous scratching behavior and skin damage, increasing mechanical itch but not chemical itch [18]. On the other hand, Gao et al. revealed that NPY and Y1 receptor agonists reduced the duration of scratching induced by compound 48/80 and histamine other than chloroquine [49]. A recent study showed that chemogenetically activating NPY^+^ neurons reduced pruritogen-evoked itch behavior tested in the leg area [27]. The differences in these findings possibly result from approaches for targeting neurons (e.g. genetic manipulation vs. pharmacology, loss-of-function vs. gain-of-function, ablation vs. transient silencing) and regional differences in the itch tests (neck vs. head vs. hindlimb). In agreement with Bourane et al., 2015 and Acton et al., 2019 [18, 25], we found that activation of persistent NPY^+^ neurons at the thoracic spinal cord level preferentially reduced mechanical itch rather than chemical itch on the neck area (Fig. 4). Furthermore, we demonstrated that spinal NPY^+^ neurons receive monosynaptic inputs from VGLUT3^+^ neurons (Fig. 3), and the antagonist of Y1R abolished DRG^VGLUT3^ -mediated inhibition of mechanical itch (Fig. 5).

In summary, our data demonstrates that optogenetic activation of sensory neurons expressing *VGLUT3* can inhibit both chemical and mechanical itch/alloknesis. These inhibitory effects are mediated through interactions with spinal DYN^+^ and NPY^+^ neurons. These findings not only shed new light on the neural basis of touch inhibition of itch, but also offer valuable scientific groundwork for the future circuit-specific therapeutic advancements for distinct forms of itch.

## Materials and methods

### Animals

All animal experiments were conducted in a double-blind manner and approved by the Animal Care and Use Committee of the University of Science and Technology of China. Mice were maintained with 12 h dark-light cycle at room temperature (23–25 °C) and *ad libitum* access to food and water. *Slc17a8*^*Cre*^ (*VGLUT3*^*Cre*^, RRID: IMSR_JAX: 028534), *ROSA*^*ChR2−EYFP*^ (*Ai32*, RRID: IMSR_JAX: 024109), *DYN*^*Cre*^ (027958: RRID: IMSR_JAX: 027958) and *ROSA*^*tdTomato*^ (*Ai14*, RRID: IMSR_JAX: 007914) mice were purchased from the Jackson Laboratory. *NPY*^*Cre*^ (RRID: IMSR_JAX: 027958) mice were gifted by Dr. Cheng Zhan (USTC, China). To generate *VGLUT3*^*Cre*^; *ROSA*^*ChR2−EYFP*^ mice (referred to as *VGLUT3*^*Cre*^*-ChR2* mice), *VGLUT3*^*Cre*^ mice were crossed with *ROSA*^*ChR2−EYFP*^ mice. To generate *VGLUT3*^*Cre*^; *DYN*^*Cre*^; *ROSA*^*ChR2−EYFP*^ mice (referred to as *VGLUT3*^*cre*^*/DYN*^*cre*^*-ChR2* mice), *VGLUT3*^*Cre*^ mice were crossed with *ROSA*^*ChR2−EYFP*^ mice, and the resulting first-generation offspring were then crossed with *DYN*^*Cre*^ mice to obtain the final *VGLUT3*^*cre*^*/DYN*^*cre*^*-ChR2* mice. To generate *DYN*^*Cre*^; *ROSA*^*tdTomato*^ mice (referred to as *DYN*^*tdTomato*^ mice), *DYN*^*Cre*^ mice were crossed with *ROSA*^*tdTomato*^ mice. Both male and female mice were included in the study.

### Immunofluorescent staining

The mice were deeply anesthetized with isoflurane and cardiac perfused with ice-cold PBS and 4% paraformaldehyde. Spinal cord, dorsal root ganglia (DRG) and skin were dissected and post-fixed in 4% PFA overnight at 4 °C, cryoprotected in 30% sucrose (w/v) in PBS, tissues were dehydrated and embedded in OCT (SAKURA), then sectioned (spinal cord 25 μm, DRG 15 μm, skin 15 μm) by a cryostat (Leica CM1950). The sections were washed PBS, blocked with blocking solution (Beyotime, P0103) for 1 h, and then incubated with primary antibodies overnight at 4 °C. The following primary antibodies were used in this study: rabbit anti-TH (1:1000, Millipore, Cat#AB152, RRID: AB_390204), rabbit anti-CGRP (1:1000, Sigma, Cat#C8198, RRID: AB_390204), rabbit anti-NF200 (1:1000, Sigma, Cat#N4142, RRID: AB_477272), Alexa-568-conjugated IB4 (1:500, Thermo Fisher Scientific, Cat#I21412), rabbit anti-PGP 9.5 (1:1000, Sigma, Cat#SAB4503057, RRID: AB_10761291). After washing in PBS, sections were incubated with Alexa Fluor 488-conjugated secondary antibody (1:500, Jackson ImmunoResearch Labs, Cat#111-585-003, RRID: AB_2338059) for 1 h at room temperature. Finally, tissues were washed in PBS and mounted on slides. Immunofluorescence images were acquired using a confocal microscope (Olympus, FV3000). Signals were counted from 5 to 6 slices per mouse for DRG, and at least 3 mice of each group were analyzed.

### Fluorescence in situ hybridization (FISH)

The in situ hybridization was conducted as previously described [48]. Briefly, the spinal cord sections (25 μm) were washed in DEPC-PBS, then fixed in 4% DEPC-PFA for 20 min, followed by two washes in DEPC-PBS. After digestion in PK buffer, sections were rinsed with DEPC-PBS and again fixed in 4% DEPC-PFA. After prehybridization at room temperature for 1 h, sections were hybridized with digoxigenin-labeled antisense cRNA probes for *NPY* and *DYN* overnight at 55 °C in hybridization solution. Then sections were washed in 2×SSC and PBT consecutively. Digoxigenin-labeled probes were detected using Anti-Digoxigenin-POD (1:1000, RRID: AB_514500, Roche) overnight at 4 °C. iF594-Tyramide (1:1000, G1242, Servicebio) was used to detect the primary antibody. The native EGFP fluorescence in spinal slices was quenched during hybridization at 55 °C. To recover EGFP signals, sections were incubated with an anti-GFP antibody (1:1000, RRID: AB_300798, Abcam), and then incubated with Alexa 488 goat anti-chicken secondary antibody (1:1000, RRID: AB_2636803, Abcam).

### Behavioral testing

All experimental animals underwent a 2-day acclimatization period, consisting of 30 min per day, prior to behavioral testing. The experimenter conducting the tests was blinded to the genotype and treatment conditions. Both male and female mice were included in the study.

### Laser sources and light guides

To investigate the suppressive effects of optogenetic manipulation on pruriceptive signaling, *VGLUT3*^*cre*^*-ChR2* mice were acclimated in a standardized plexiglass chamber (20 × 20 × 15 cm) for 30 min prior to stimulation. The optical delivery system incorporated a handheld fiber optic probe (core diameter 1.0 mm) with 1 cm distance, consisting of two distinct laser modules: a 473 nm blue laser (QAXK-LASER) coupled to a 1.0 mm core diameter optical fiber (Inper) for selective ChR2 activation, and a 594 nm yellow laser (QAXK-LASER) serving as wavelength-matched control. Cutaneous illumination was administered to either the cervical dermatome (nape region) or auricular area using pulsed light parameters: 5 ms pulse width at 20 Hz frequency, with calibrated irradiance of 20 mW/mm² sustained for 30 min. Photonic flux density was verified using a calibrated thermopile power meter (Sanwa LP1-TC) positioned at the tissue interface prior to each experimental session.

### Chemical itch test

To test the acute chemical itch, mice had the fur on their napes shaved at least 5 days prior to behavioral testing. Mice were given a 30 min acclimation period before testing. The pruritogens histamine (500 µg, sigma) and chloroquine (200 µg, sigma) were dissolved in saline and administered intradermally at the nape in a 50 µL volume [67]. The scratch behavior was recorded by camera for the next 30 min and quantified by the count of scratching episodes. A bout of scratching was defined as continuous scratching of the injected site by the hind paw until the mouse placed its hind paw in its mouth or on the chamber floor.

### Acute mechanical itch test

To test mechanical itch, the fur behind the ears was shaved at least 5 days prior to behavioral testing. On the test day, after a 30 min acclimatization period in the testing chambers, mice received 10 separate innocuous mechanical stimuli, each lasting approximately 1 s, delivered using a von Frey filament (0.07 g) with intervals of 3–5 s between stimuli. The overall mechanical itch score was calculated based on hindlimb scratching responses observed during the 10 trials.

### Histamine-induced alloknesis

To test mechanical sensitization, a histamine-induced alloknesis test was conducted. Mice had their nape fur shaved at least 5 days prior to behavioral testing. On the test day, after a 30 min acclimatization period in the testing chambers, a low-dose of histamine (50 µg/10 µL in saline, Sigma) was intradermally administered at the nape. After 30 min following histamine injection, mice received three separate innocuous mechanical stimuli as described above, at randomly selected sites oriented radially 0.5–1 cm away from the injection site. This testing was repeated for 30 min at 5 min intervals (from 30 to 60 min after injection, totaling 21 stimuli). The overall alloknesis score was calculated based on hindlimb scratching responses observed during the 21 trials.

### Itch-induced conditioned place aversion (CPA) test

A black and white chamber (200 mm × 100 mm × 100 mm) partitioned by a 40 mm × 40 mm hole was employed for CPA testing. All animals underwent a two-day habituation phase with the custom-made apparatus, where they were allowed to explore it for 30 min each day.

The CPA experiment spanned five days and was divided into three phases: the pre-stimulation phase (day 1), the stimulation phase (day 2 to day 4), and the post-stimulation phase (day 5). During the pre-stimulation phase, mice had free access to the apparatus for 15 min. In the stimulation phase, itch stimuli were administered twice daily (morning and afternoon) for three consecutive days. In the morning sessions of day 2 and day 4, mice received saline injections (i.d.) at the nape and were placed in the white chamber for 30 min, designated as the ‘itch-unpaired’ compartment. In the afternoon sessions of the same days, the mice were injected with histamine (i.d.) and placed in the black chamber for 30 min, designated as the ‘itch-paired’ compartment. On day 3, histamine was administered in the morning (black chamber), and saline was administered in the afternoon (white chamber). Animals were restricted to access only one chamber during each conditioning trial. The post-stimulation phase allowed mice to explore the apparatus freely for 15 min. The time spent in the black chamber was recorded on both day 1 and day 5. CPA data were analyzed by measuring the time (in seconds) spent in the black chamber during the pre-test and post-test for individual animals. The normalized CPA score was calculated as the time of post-test divided by pre-test for in the black chamber.

In CPA experiments involving optogenetic manipulations, yellow or blue light stimuli were used to activate VGLUT3-lineage sensory nerves during each conditioning session. In CPA experiments involving chemogenetic manipulations, Clozapine N-oxide (CNO) (Cat#A3317, APExBIO) was injected (i.p.) 30 min before each conditioning session.

### Drug administration

The nor-Binaltorphimine (nor-BNI) (Cat#AB120078, Abcam) and BMS193885 (Cat#HY-120619, MCE) were dissolved in saline. In contrast, CNO was dissolved in PBS. CNO was administered via intraperitoneal (i.p.) injection at a dosage of 2 mg/kg for the chemogenetic activation of DYN^+^ or NPY^+^ neurons. CNO injections were performed 30 min prior to behavioral testing. BMS193885 (1 µg in 10 µL) was administered through intrathecal (i.t.) injection, and behavioral testing was conducted 15–45 min after administration. Mice were pre-treated with nor-BNI (1 µg in 10 µL) 24 h before experimentation.

### Intraspinal stereotaxic injection

#### Chemogenetic stimulation of spinal DYN^+^ or NPY^+^ neurons

Intraspinal injections were conducted following a modified version as previously described [48]. Briefly, mice were anesthetized with 2.5% isoflurane, and their limbs were secured with tapes. A small incision was made along the neck to expose the spaces between the laminae of C3–C4 and C5–C6 vertebrae. Small incisions were made in the dura on both sides of the midline within each of these two spaces. Next, 500 nL of the rAAV-EF1α-DIO-hM3D(Gq)-mCherry-WPREs (5.33E + 12 vg/mL, BrainVTA, PT-0042) virus or rAAV-EF1α-DIO-mCherry-WPRE-pA (5.19E + 12 vg/mL, BrainVTA, PT-0013) control virus were injected on the four sites through each of these two incisions in the dura at a depth of 300 μm below the spinal cord surface and 400 μm lateral to the midline. To minimize leakage, the pipette was maintained for 5 min after the completion of each injection. Injections were made by using a syringe pump (RWD Life Science, China) and a 10 µL Hamilton syringe attached a glass micropipette at a rate of 30 nL/min. Behavioral testing was conducted four weeks after full recovery from the surgery.

#### Anterograde tracing of VGLUT3-lineage neurons

For anterograde tracing of VGLUT3-lineage neurons, 5 µL of rAAV-CAG-DIO-WGA-FLP-WPREs (5.43E + 12 vg/mL, BrainVTA, PT-0557) virus were intrathecally injected into *VGLUT3*^*cre*^ mice. After two weeks, 300 nL of rAAV-fDIO-EGFP-WPREs (5.08E + 12 vg/mL, BrainVTA, PT-0435) virus were bilaterally injected into the lumbar spinal cord. Four weeks later, the fluorescence in situ hybridization experiment was performed with *DYN/NPY-*RNA probe.

#### Rabie virus tracing

For retrograde tracing of SC^DYN^ and SC^NPY^ neurons, helper viruses containing rAAV2/9-Ef1α-DIO-RVG-WPRE-pA (5.29E + 12 vg/mL, BrainVTA, PT-0061) and rAAV2/9-Ef1α-DIO-EGFP-2a-TVA-WPRE-pA (5.56E + 12 vg/mL, BrainVTA, PT-0062, 1:2, 200 nL) were co-injected into the lumbar spinal cord of *DYN*^*cre*^ mice or *NPY*^*cre*^ mice. After two weeks, 200 nL RV-EnvA-ΔG-dsRed (2E + 8 IFU/mL, BrainVTA, R01002) was injected into the same site in the spinal cord. One week after the final injection, mice were sacrificed, and the DRG were prepared for immunofluorescence staining.

#### Labeling spinal NPY^+^ neurons

To label spinal NPY^+^ neurons, 300 nL of rAAV-NPY-mCherry-WPRE-hGH-pA (A rAAV plasmid backbone containing the woodchuck hepatitis virus post-transcriptional regulatory element (WPRE) and the bovine growth hormone polyadenylation sequence (bGHpA) flanked by AAV9 inverted terminal repeats was used to drive the cDNA encoding enhanced mCherry fluorescent protein (mCherry) under the control of the CMV enhancer/neuropeptide Y promoter) (6.05E + 12 vg/mL, BrainVTA, PT-1216, diluted 1:10 in PBS) virus were injected into *VGLUT3-ChR2* mice lumbar spinal cord. The electrophysiological recording was performed 3 weeks after the virus injection.

### Electrophysiology

#### Spinal cord slice preparation

Procedures for electrophysiological recordings followed those described in previous publications [48]. For slice preparation, mice were deeply anesthetized with isoflurane and intracardially perfused with 20 mL of ice-cold oxygenated N-Methyl-D-Glutamine (NMDG)-artificial cerebrospinal fluid (ACSF) containing the following (in mM): 93 N-Methyl-D-Glutamine (NMDG), 2.5 KCl, 1.2 NaH_2_PO4, 30 NaHCO_3_, 20 HEPES, 25 glucose, 2 thiourea, 5 Na-ascorbate, 3 Na-pyruvate, 0.5 CaCl_2_, 10 MgSO_4_, and 3 glutathione (pH 7.3–7.4, 300–305 mOsm). The lumbar spinal cord was quickly removed and transferred into ice-cold NMDG-ACSF, followed by cutting into parasagittal spinal cord slices with attached dorsal roots using a vibratome (VT1200S, Leica). The spinal slices were then transferred to 32 °C NMDG-ACSF for 10 min and subsequently to 25 °C oxygenated HEPES-ACSF, which contained the following (in mM): 92 NaCl, 2.5 KCl, 1.2 NaH_2_PO_4_, 30 NaHCO_3_, 20 HEPES, 25 glucose, 2 thiourea, 5 Na-ascorbate, 3 Na-pyruvate, 2 CaCl_2_, 2 MgSO_4_, and 3 glutathione (300–305 mOsm) for at least 1 h. Slices were then transferred to a recording chamber and continuously perfused with room temperature oxygenated recording ACSF for patch clamp recording.

#### Patch-clamp recordings

Whole-cell recording experiments were conducted as described previously [48]. The recording ACSF composition was as follows (in mM): 125 NaCl, 2.5 KCl, 2 CaCl_2_, 1 MgCl_2_, 1.25 NaH_2_PO_4_, 26 NaHCO_3_, 25 d-glucose, 1.3 sodium ascorbate, and 3.0 sodium pyruvate, with an osmolarity of 300–305 mOsm. The internal solution contained (in mM): 130 potassium gluconates, 5 KCl, 4 Na_2_ATP, 0.5 Na-GTP, 20 HEPES, and 0.5 EGTA, with a pH adjusted to 7.28 using KOH, and an osmolarity of 280–300 mOsm. Electrophysiological recording data were acquired using pClamp 10.0 software with a MultiClamp 700B patch-clamp amplifier (Molecular Devices) and Digidata 1550B (Molecular Devices). Responses were low-pass filtered on-line at 2 kHz and digitized at 5 kHz.

To optogenetically activate VGLUT3^+^ sensory neurons, blue light stimulation was delivered to the DRG through a 200 μm optical fiber (Inper, China) connected to a laser (473 nm, QAXK-LASER). VGLUT3^+^ neurons were stimulated with pulses of blue light (473 ± 5 nm wavelength). After identifying NPY-mCherry neurons, light-evoked excitatory postsynaptic currents (L-eEPSCs) were recorded at a holding potential of -70 mV. To examine the monosynaptic connection between VGLUT3^+^ neurons and spinal NPY^+^ neurons, monosynaptic VGLUT3^+^ fiber inputs were identified by the absence of failures in response to 10 stimuli at 1 Hz, and jitter was calculated as the standard deviation of the latency values of seven consecutive L-eEPSCs at 0.05 Hz. TTX (1 µM) was used to block action potential-based synaptic transmission, and both TTX (1 µM) and 4-AP (300 µM) were used to restore monosynaptic current.

### Statistical analysis

The data were subjected to analysis using GraphPad Prism, Olympus FV10-ASW 4.0a Viewer, ImageJ, and Clampfit software. All experiments and data analyses were conducted in a double-blind manner, including immunofluorescence, behavioral analyses, and electrophysiology. Normality was assessed using the Shapiro-Wilk test. If the data were normally distributed, paired t-tests and unpaired t-tests were appropriately applied. In cases where normality was violated, the data were analyzed using the Wilcoxon signed-rank test for paired tests and the Mann-Whitney U test for unpaired tests. *P* < 0.05 was considered as a significant change.

## Supplementary Information

Below is the link to the electronic supplementary material.

Supplementary Material 1

## Data Availability

No datasets were generated or analysed during the current study.

## Abbreviations

ACSF: Artificial Cerebrospinal Fluid
AP: Action Potential
C-LTMRs: C-Low-Threshold Mechanoreceptors
CPA: Conditioned Place Aversion
DRG: Dorsal Root Ganglia
DYN: Dynorphin
EGFP: Enhanced Green Fluorescent Protein
EPSC: Excitatory Postsynaptic Current
EPSP: Excitatory Postsynaptic Potential
FISH: Fluorescence in situ Hybridization
GINIP: Gαi-Interacting Protein
KOR: Kappa Opioid Receptor
NPY: Neuropeptide Y
SC: Spinal Cord
TH: Tyrosine Hydroxylase
VGLUT3: Vesicular Glutamate Transporter 3
WDS: Wet Dog Shake
