# Supplementary material for: Identification of VGLUT3-expressing LTMRs-recruited spinal circuits for itch inhibition
Source: Mol Brain. 2025 Sep 30;18:74. doi: 10.1186/s13041-025-01245-3 (PMC12487289; doi:10.1186/s13041-025-01245-3)
Supplement: Supplementary file 1 — Supplementary Material 1 [file 13041_2025_1245_MOESM1_ESM.pdf]

Identification of VGLUT3-expressing LTMRs-recruited spinal circuits for itch inhibition

Xiaojing Su,[1, #] Liangbiao Wang,[1, 2, #] Xiaoqing Liu,[3] and Yan Zhang[1, *]

Supplementary information

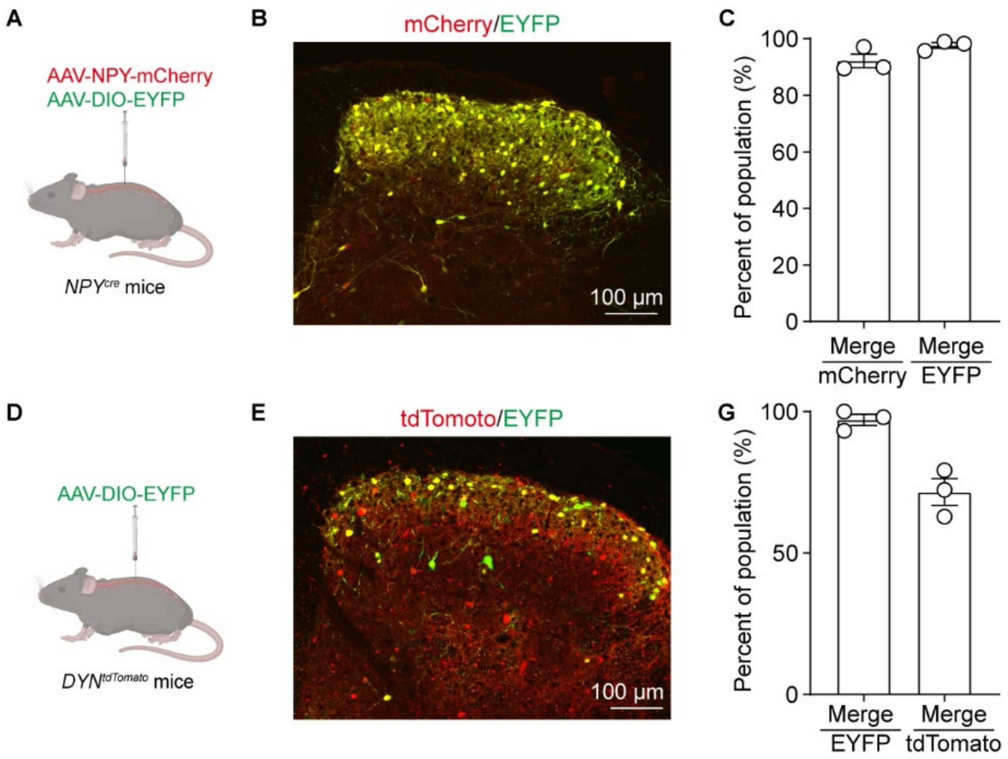

Supplementary Figure 1. Viral specificity validation for labeling SCNPY and SCDYN neurons.

A Schematic of intraspinal injection of AAV-NPY-mCherry and AAV-DIO-EYFP in *NPY^{Cre}* mice. **B-C** Representative image showing co-localization of mCherry with EYFP-expressing neurons in *NPY^{Cre}* mice and statistical data (n = 3 mice). Scale bar, 100 µm. **D** Schematic of intraspinal injection of AAV-DIO-EYFP in *DYN^{tdTomato}* mice. **E-G** Representative image showing co-localization of tdTomato with EYFP-expressing neurons in *DYN^{tdTomato}* mice and statistical data (n = 3 mice). Scale bar, 100 µm. Data are represented as mean with SEM.

A

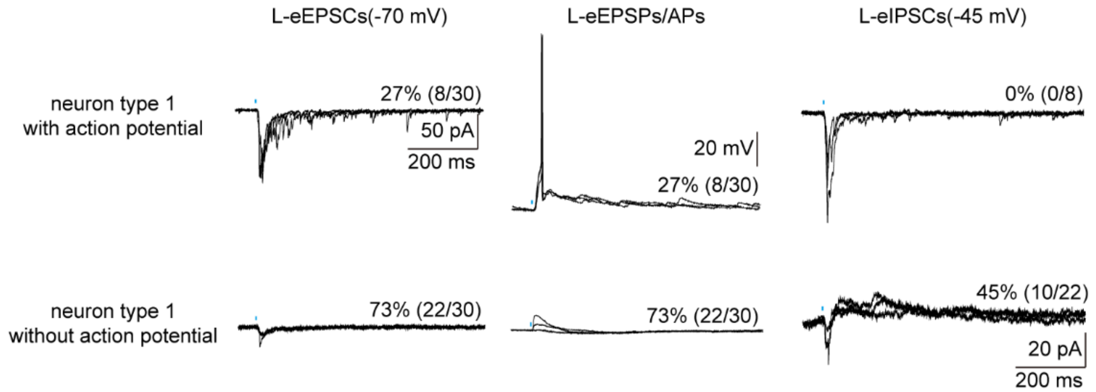

Supplementary Figure 2. Two types of light-evoked synaptic responses in spinal cord neurons in *VGLUT3^{Cre}-ChR2* Mice.

A Two distinct categories of light-evoked synaptic responses of spinal dorsal horn neurons in *VGLUT3^{Cre}-ChR2* mice.
